# A note on some identities of derangement polynomials

**DOI:** 10.1186/s13660-018-1636-8

**Published:** 2018-02-17

**Authors:** Taekyun Kim, Dae San Kim, Gwan-Woo Jang, Jongkyum Kwon

**Affiliations:** 1 Department of Mathematics, College of Science, Tianjin Polytechnic University, Tianjin, China; 2 Department of Mathematics, Kwangwoon University, Seoul, Republic of Korea; 3 Department of Mathematics, Sogang University, Seoul, Republic of Korea; 4 Department of Mathematics Education and ERI, Gyeongsang National University, Jinju, Republic of Korea

**Keywords:** 05A19, 05A40, 11B73, 11B83, Derangement numbers, Derangement polynomials, *r*-derangement numbers, *r*-derangement polynomials, Umbral calculus

## Abstract

The problem of counting derangements was initiated by Pierre Rémond de Montmort in 1708 (see Carlitz in Fibonacci Q. 16(3):255–258, 1978, Clarke and Sved in Math. Mag. 66(5):299–303, 1993, Kim, Kim and Kwon in Adv. Stud. Contemp. Math. (Kyungshang) 28(1):1–11 2018. A derangement is a permutation that has no fixed points, and the derangement number $d_{n}$ is the number of fixed-point-free permutations on an *n* element set. In this paper, we study the derangement polynomials and investigate some interesting properties which are related to derangement numbers. Also, we study two generalizations of derangement polynomials, namely higher-order and *r*-derangement polynomials, and show some relations between them. In addition, we express several special polynomials in terms of the higher-order derangement polynomials by using umbral calculus.

## Introduction

Let $\mathbb{C}$ be the complex number field, and let $\mathcal{F}$ be the set of all formal power series in the variable *t* with coefficients in $\mathbb{C}$:

1.1 $$ \begin{aligned}[b] \mathcal{F}= \Biggl\{ f(t)= \sum _{k=0}^{\infty}a_{k} \frac {t^{k}}{k!}\Bigm|a_{k} \in\mathbb{C} \Biggr\} . \end{aligned} $$

Let $\mathbb{P} = \mathbb{C}[x]$, and let $\mathbb{P}^{*}$ be the vector space of all linear functionals on $\mathbb{P}$. We denote the action of a linear functional $L \in\mathbb{P}^{*}$ on polynomials $p(x) \in\mathbb{P}$ by $\langle L\mid p(x)\rangle$, and it is known that vector space operations on $\mathbb{P}^{*}$ are defined by

1.2 $$ \bigl\langle L+M\mid p(x)\bigr\rangle = \bigl\langle L\mid p(x) \bigr\rangle + \bigl\langle M\mid p(x)\bigr\rangle , \qquad \bigl\langle c L\mid p(x)\bigr\rangle = c \bigl\langle L\mid p(x)\bigr\rangle , $$

where *c* is a complex constant (see [3–5]).

For $f(t)= \sum_{k=0}^{\infty}a_{k} \frac{t^{k}}{k!}$, we define a linear functional on $\mathbb{P}$ by setting

1.3 $$ \bigl\langle f(t)\mid x^{n}\bigr\rangle = a_{n} \quad(n\geq0)\ (\mbox{see [6, 7]}). $$

From (1.3), we note that

1.4 $$ \bigl\langle t^{k}\mid x^{n}\bigr\rangle = n! \delta_{n,k} \quad(n,k \geq0) \ (\mbox{see [8]}), $$

where $\delta_{n,k}$ is the Kronecker symbol.

The order $o(f(t))$ of a power series $f(t)(\neq0) \in\mathcal{F}$ is the smallest integer *k* such that the coefficients of $t^{k}$ do not vanish. For $f(t), g(t) \in\mathcal{F}$, with $o(f(t))=1$ and $o(g(t))=0$, there exists a unique sequence $S_{n}(x)$ of polynomials such that $\langle g(t) f(t)^{k} | S_{n}(x)\rangle = n! \delta_{n,k}$ for $n,k \geq 0$ (see [5, 8]). The sequence $S_{n}(x)$ is called the Sheffer sequence for $(g(t), f(t))$, which is denoted by $S_{n}(x) \sim(g(t), f(t))$. It is known that $S_{n}(x) \sim(g(t),f(t))$ if and only if

1.5 $$ \begin{aligned}[b] \frac{1}{g(\bar{f}(t))}e^{x\bar{f}(t)} = \sum _{n=0}^{\infty}S_{n}(x) \frac{t^{n}}{n!}, \end{aligned} $$

where $\bar{f}(t)$ is the compositional inverse of $f(t)$ with

1.6 $$ f\bigl(\bar{f}(t)\bigr) = \bar{f}\bigl(f(t)\bigr) = t \quad( \mbox{see [8, 9]}). $$

For $f(t) \in\mathcal{F}$ and $p(x) \in\mathbb{P}$, by (1.4), we get

1.7 $$ f(t) = \sum_{k=0}^{\infty}\bigl\langle f(t) \vert x^{k}\bigr\rangle \frac {t^{k}}{k!}, \qquad p(x) = \sum_{k=0}^{\infty}\bigl\langle t^{k} \vert p(x)\bigr\rangle \frac{x^{k}}{k!} \quad(\mbox{see [9]}). $$

From (1.7), we note that

1.8 $$ p^{(k)}(0) = \bigl\langle t^{k} \vert p(x)\bigr\rangle = \bigl\langle 1 \vert p^{(k)}(x)\bigr\rangle \quad(k \geq0), $$

where $p^{(k)}(x) = (\frac{d}{dx})^{k} p(x)$.

From (1.8), we easily get

1.9 $$ t^{k}p(x) = p^{(k)}(x), \qquad e^{yt}p(x) = p(x+y), \qquad \bigl\langle e^{yt} \vert p(x) \bigr\rangle = p(y) \quad(\mbox{see [9]}). $$

Let $S_{n}(x) \sim(g(t), f(t))$ and $r_{n}(x) \sim(h(t), l(t))$ ($n \geq0$). Then we have

1.10 $$ \begin{aligned}[b] S_{n}(x) = \sum _{m=0}^{n} C_{n,m}r_{m}(x) \quad (n \geq0)\ (\mbox{see [8, 9]}), \end{aligned} $$

where

1.11 $$ \begin{aligned}[b] C_{n,m} = \frac{1}{m!} \biggl\langle \frac{h(\bar{f}(t))}{g(\bar {f}(t))}l\bigl(\bar{f}(t)\bigr)^{m} \Big\vert x^{n} \biggr\rangle \quad(n,m \geq0). \end{aligned} $$

For $u(\neq1) \in\mathbb{C}$, the Frobenius-Euler numbers are defined by the generating function

1.12 $$ \frac{1-u}{e^{t}-u} = \sum_{n=0}^{\infty} H_{n}(u) \frac{t^{n}}{n!} \quad(\mbox{see [10--12]}). $$

When $u=-1$, $H_{n}(-1)=E_{n}$ are the ordinary Euler numbers.

The Bernoulli polynomials are given by

1.13 $$ \frac{t}{e^{t}-1} e^{xt} = \sum _{n=0}^{\infty} B_{n}(x) \frac {t^{n}}{n!} \quad(\mbox{see [3, 12, 13]}). $$

When $x=0$, $B_{n} = B_{n}(0)$ are the Bernoulli numbers.

We know that the Euler polynomials are defined by

1.14 $$ \frac{2}{e^{t}+1} e^{xt} = \sum _{n=0}^{\infty} E_{n}(x) \frac {t^{n}}{n!} \quad(\mbox{see [10, 11]}). $$

When $x=0$, $E_{n} = E_{n}(0)$ are the Euler numbers.

The falling factorial sequence is defined as

1.15 $$ (x)_{0} = 1, (x)_{n} = x(x-1) \cdots(x-n+1) \quad (n \geq1). $$

The Stirling numbers of the first kind are defined by

1.16 $$ (x)_{n} = \sum_{l=0}^{n} S_{1}(n,l)x^{l} \quad (n \geq0)\ (\mbox{see [8]}), $$

and the Stirling numbers of the second kind are given by

1.17 $$ x^{n} = \sum_{l=0}^{n} S_{2}(n,l) (x)_{l}\quad (n \geq0) \ (\mbox{see [8, 14, 15]}). $$

The Stirling numbers of the second kind are also given by the exponential generating function (see [8, p.59])

1.18 $$ \frac{1}{k!} \bigl(e^{t} -1\bigr)^{k}= \sum_{n=k}^{\infty} S_{2}(n,k) \frac{t^{n}}{n!}. $$

It is well known that the Bell polynomials are defined by the generating function

1.19 $$ e^{x(e^{t}-1)}=\sum_{n=0}^{\infty}\mathrm{Bel}_{n}(x) \frac{t^{n}}{n!} \quad (\mbox{see [9]}). $$

When $x=1$, $\mathrm{Bel}_{n} = \mathrm{Bel}_{n}(1)$ ($n \geq0$) are the Bell numbers.

From (1.19), we have

1.20 $$ \mathrm{Bel}_{n}(x) = \sum_{k=0}^{n} S_{2}(n,k) x^{k} \quad (n \geq0) \ (\mbox{see [9]}). $$

A derangement is a permutation that has no fixed points. The derangement number $d_{n}$ is the number of fixed-point-free permutations on an *n* element set (see [1–3]). The problem of counting derangements was initiated by Pierre Rémond de Montmort in 1708 (see [1–3]). The first few terms of the derangement number sequence $\{d_{n}\}_{n=0}^{\infty}$ are $d_{0} =1$, $d_{1} = 0$, $d_{2} = 1$, $d_{3} = 2$, $d_{4} = 9$, $d_{5} = 44$, $d_{6} = 265$, $d_{7} = 1854$, … .

Indeed, $d_{n}$ is given by the closed form formula:

1.21 $$ d_{n} = n! \sum_{k=0}^{n} \frac{(-1)^{k}}{k!} \quad(\mbox{see [3]}). $$

From (1.21), we note that the generating function of derangement numbers is given by

1.22 $$ \frac{1}{1-t} e^{-t} = \sum _{n=0}^{\infty} d_{n} \frac {t^{n}}{n!}\quad(\mbox{see [9]}). $$

By using (1.22), it is not difficult to show that

1.23 $$ d_{m} = (m-1) (d_{m-1} + d_{m-2}) \quad (m \geq2),\qquad d_{0} = 1,\qquad d_{1}=0, $$

and

1.24 $$ d_{m} = m d_{m-1} + (-1)^{m} \quad (m \geq1),\qquad d_{0} = 1 \quad (\mbox{see [1--3]}). $$

For $r \in\mathbb{N}$, the derangement numbers $d_{n}^{(r)}$ of order *r* ($n \geq0$), are defined by the generating function

1.25 $$ \biggl(\frac{1}{1-t} \biggr)^{r} e^{-t} = \sum_{n=0}^{\infty}d_{n}^{(r)} \frac{t^{n}}{n!}\quad(\mbox{see [3]}). $$

The umbral calculus comes under the heading of combinatorics, the calculus of finite differences, the theory of special functions, and formal solutions to differential equations. Also, formal power series play a predominant role in the umbral calculus. In this paper, we study the derangement polynomials and investigate some interesting properties which are related to derangement numbers. Further, we study two generalizations of derangement polynomials, namely higher-order and *r*-derangement polynomials, and show some relations between them. In addition, we express several special polynomials in terms of the higher-order derangement polynomials by using umbral calculus.

## Some identities of derangement polynomials arising from umbral calculus

Now, we define the derangement polynomials by

2.1 $$ \sum_{n=0}^{\infty} d_{n}(x) \frac{t^{n}}{n!} = \frac{1}{1-t} e^{xt}. $$

We note here that, for $x=-1$, $d_{n} = d_{n}(-1)$ are the derangement numbers.

We observe that

2.2 $$ \begin{aligned}[b] \frac{1}{1-t} e^{xt} & = e^{-\log(1-t)} e^{xt} = \Biggl(\sum_{m=0}^{\infty } (-1)^{m} \frac{1}{m!}\bigl(\log(1-t)\bigr)^{m} \Biggr) \Biggl(\sum_{l=0}^{\infty} x^{l} \frac {t^{l}}{l!} \Biggr) \\ & = \Biggl(\sum_{k=0}^{\infty} \Biggl(\sum _{m=0}^{k} (-1)^{k-m} S_{1}(k,m) \Biggr) \frac {t^{k}}{k!}\Biggr) \Biggl(\sum _{l=0}^{\infty} x^{l}\frac{t^{l}}{l!} \Biggr) \\ & = \sum_{n=0}^{\infty} \Biggl(\sum _{k=0}^{n} \sum_{m=0}^{k} \binom {n}{k} (-1)^{k-m} S_{1}(k,m) x^{n-k} \Biggr) \frac{t^{n}}{n!}. \end{aligned} $$

By (2.1) and (2.2), we get

2.3 $$ d_{n}(x) = \sum_{k=0}^{n} \sum_{m=0}^{k} \binom{n}{k} (-1)^{k-m} S_{1}(k,m) x^{n-k} \quad (n \geq0), $$

and

2.4 $$ d_{n} = \sum_{k=0}^{n} \sum_{m=0}^{k} \binom{n}{k} (-1)^{n-m} S_{1}(k,m) \quad (n \geq0). $$

Therefore we obtain the following lemma.

### Lemma 2.1

*For* $n \geq0$, *we have*

$$ d_{n}(x) = \sum_{k=0}^{n} \sum _{m=0}^{k} \binom{n}{k} (-1)^{k-m} S_{1}(k,m) x^{n-k} $$

*and*

$$ d_{n} = \sum_{k=0}^{n} \sum _{m=0}^{k} \binom{n}{k} (-1)^{n-m} S_{1}(k,m). $$

From (2.1), we have

2.5 $$ \begin{aligned}[b] \sum_{n=0}^{\infty} d_{n}(x) \frac{t^{n}}{n!} & = \biggl(\frac{1}{1-t} e^{-t}\biggr) e^{(x+1)t} \\ & = \Biggl(\sum_{m=0}^{\infty} d_{m} \frac{t^{m}}{m!}\Biggr) \Biggl(\sum_{l=0}^{\infty } (x+1)^{l} \frac{t^{l}}{l!} \Biggr) \\ & = \sum_{n=0}^{\infty}\Biggl(\sum _{m=0}^{n} \binom{n}{m} d_{m} (x+1)^{n-m} \Biggr) \frac{t^{n}}{n!}. \end{aligned} $$

Therefore, we obtain the following proposition.

### Proposition 2.2

*For* $n \geq0$, *we have*

$$ d_{n}(x) = \sum_{m=0}^{n} \binom{n}{m} d_{m} (x+1)^{n-m} = (d+x+1)^{n}, $$

*with the usual convention about replacing* $d^{n}$ *by* $d_{n}$.

From Proposition 2.2, we have

2.6 $$ \frac{d}{dx} d_{n}(x) = \frac{d}{dx}(d+x+1)^{n} = n (d+x+1)^{n-1} = n d_{n-1}(x) \quad (n \geq1). $$

By (1.5) and (2.1), we get

2.7 $$ d_{n}(x) \sim(1-t, t). $$

That is, $d_{n}(x)$ ($n \geq0$) is an Appell sequence.

Now, we note that

2.8 $$ \begin{aligned}[b] \sum_{n=0}^{\infty} d_{n}(x) \frac{t^{n}}{n!} & = \biggl(\frac{1}{1-t} e^{t}\biggr) e^{(x-1)t} \\ & = \sum_{n=0}^{\infty} \Biggl(\sum _{l=0}^{n} \binom{n}{l} a_{l} (x-1)^{n-l} \Biggr) \frac{t^{n}}{n!}, \end{aligned} $$

where $a_{n}$ are the arrangement numbers defined by

2.9 $$ \frac{1}{1-t} e^{t} = \sum _{n=0}^{\infty} a_{n} \frac{t^{n}}{n!}. $$

Replacing *t* by $e^{t}-1$ in (2.1), we get

2.10 $$ \begin{aligned}[b] \sum_{k=0}^{\infty} d_{k}(x) \frac{1}{k!} \bigl(e^{t} -1 \bigr)^{k} & = \biggl(\frac {1}{2-e^{t}} \biggr) e^{x(e^{t}-1)} \\ & = \Biggl(\sum_{l=0}^{\infty} H_{l}(2) \frac{t^{l}}{l!}\Biggr) \Biggl(\sum _{m=0}^{\infty} \mathrm{Bel}_{m}(x) \frac{t^{m}}{m!} \Biggr) \\ & = \sum_{n=0}^{\infty} \Biggl(\sum _{l=0}^{n} \binom{n}{l} H_{l}(2) \mathrm{Bel}_{n-l}(x) \Biggr) \frac{t^{n}}{n!}. \end{aligned} $$

On the other hand,

2.11 $$ \begin{aligned}[b] \sum_{k=0}^{\infty} d_{k}(x) \frac{1}{k!} \bigl(e^{t} -1 \bigr)^{k} & = \sum_{k=0}^{\infty} d_{k}(x) \sum_{n=k}^{\infty} S_{2}(n,k) \frac {t^{n}}{n!} \\ & = \sum_{n=0}^{\infty} \Biggl(\sum _{k=0}^{n} S_{2}(n,k) d_{k}(x) \Biggr) \frac {t^{n}}{n!}. \end{aligned} $$

Therefore, by (2.10) and (2.11), we obtain the following theorem.

### Theorem 2.3

*For* $n \geq0$, *we have*

$$ \sum_{k=0}^{n} \binom{n}{k} H_{k}(2) \mathrm{Bel}_{n-k}(x) = \sum_{k=0}^{n} S_{2}(n,k) d_{k}(x). $$

For $S_{n}(x) \sim(g(t), t)$, from (1.5) we have

2.12 $$ \frac{1}{g(t)} e^{xt} = \sum _{n=0}^{\infty} S_{n}(x) \frac{t^{n}}{n!}. $$

Thus, by (2.12), we get

2.13 $$ \frac{1}{g(t)} x^{n} = S_{n}(x)\quad (n \geq0)\quad \Longleftrightarrow\quad S_{n}(x) \sim\bigl(g(t), t\bigr). $$

In (2.13), we take $g(t)= 1-t$, then we have

2.14 $$ \frac{1}{1-t}x^{n} = d_{n}(x)\quad (n \geq0), \qquad t\, d_{n}(x) = n \, d_{n-1} (x) \quad (n \geq1). $$

Now, we observe that

2.15 $$ \begin{aligned}[b] d_{n}(x+y) & = (d+x+y+1)^{n} = \sum_{l=0}^{n} \binom{n}{l} (d+x+1)^{l} y^{n-l} \\ & = \sum_{l=0}^{n} \binom{n}{l} d_{l}(x) y^{n-l} \quad (n \geq0). \end{aligned} $$

From (2.15), we note that

2.16 $$ \begin{aligned}[b] \frac{1}{n+1} \bigl(d_{n+1} (x+y) - d_{n+1} (x) \bigr) & = \frac{1}{n+1} \sum_{k=1}^{n+1} \binom{n+1}{k} d_{n+1-k} (x) y^{k} \\ & = \sum_{k=1}^{n+1} \frac{n(n-1) \cdots(n-k+2)}{k!} d_{n+1-k} y^{k} \\ & = \sum_{k=1}^{n+1} \frac{y^{k}}{k!} t^{k-1} d_{n}(x). \end{aligned} $$

By (2.15) and (2.16), we get

2.17 $$ \begin{aligned}[b] \int_{x}^{x+y} d_{n}(u) \,du & = \sum _{k=1}^{n+1} \frac{y^{k}}{k!} t^{k-1} d_{n}(x) \\ & = \frac{1}{t} \sum_{k=1}^{n} \binom{n}{k} d_{n-k} (x) y^{k} = \frac {1}{t} \bigl(e^{yt}d_{n}(x) - d_{n}(x) \bigr) \\ & = \frac{1}{t} \bigl(e^{yt} -1\bigr) d_{n}(x) \quad (n \geq0). \end{aligned} $$

From (2.17), we can derive the following equation.

2.18 $$ d_{n}(x) = \frac{t}{e^{t} -1} \int_{x}^{x+1} d_{n}(u) \,du = \frac {1}{1-t} x^{n} \quad(n \geq0). $$

### Theorem 2.4

*For* $n \geq0$, *we have*

$$ d_{n}(x) = \frac{t}{e^{t} -1} \int_{x}^{x+1} d_{n}(u) \,du = \frac {1}{1-t} x^{n}. $$

From (1.10), we have

2.19 $$ \begin{aligned}[b] \biggl\langle \frac{e^{yt}-1}{t} \Bigm|d_{n}(x) \biggr\rangle & = \biggl\langle e^{yt}-1 \Bigm|\frac{1}{n+1} d_{n+1} (x)\biggr\rangle \\ & = \biggl\langle 1\Bigm|\frac{1}{n+1} \bigl(d_{n+1} (x+y) - d_{n+1} (x) \bigr)\biggr\rangle \\ & = \frac{1}{n+1} \bigl(d_{n+1} (y) - d_{n+1} (0) \bigr) \\ & = \int_{0}^{y} d_{n}(u) \,du \quad (n \geq0). \end{aligned} $$

In particular,

2.20 $$ \begin{aligned}[b] \sum_{n=0}^{\infty} d_{n}(0) \frac{t^{n}}{n!} & = \biggl(\frac{1}{1-t} e^{-t}\biggr) e^{t} \\ & = \Biggl(\sum_{l=0}^{\infty} d_{l} \frac{t^{l}}{l!} \Biggr) \Biggl(\sum_{m=0}^{\infty} \frac{t^{m}}{m!} \Biggr) \\ & = \sum_{n=0}^{\infty} \Biggl(\sum _{l=0}^{n} \binom{n}{l} d_{l} \Biggr) \frac {t^{n}}{n!}. \end{aligned} $$

Comparing the coefficients on both sides of (1.17), we have

2.21 $$ \begin{aligned}[b] d_{n}(0) = \sum _{l=0}^{n} {n \choose l} d_{l} \quad(n \geq0). \end{aligned} $$

Therefore, we obtain the following corollary.

### Corollary 2.5

*For* $n \geq0$, *we have*

$$ \begin{aligned}[b] d_{n}(0) = \sum _{l=0}^{n} {n \choose l} d_{l} \end{aligned} $$

*and*

$$ \begin{aligned}[b] \biggl\langle \frac{e^{yt}-1}{t} \Bigm|d_{n}(x) \biggr\rangle = \int_{0}^{y} d_{n}(u) \,du. \end{aligned} $$

For $r \in\mathbb{N}$, we define the derangement polynomials of order *r* by

2.22 $$ \begin{aligned}[b] \biggl( \frac{1}{1-t} \biggr)^{r} e^{xt} = \sum_{n=0}^{\infty}d_{n}^{(r)}(x) \frac{t^{n}}{n!}. \end{aligned} $$

When $x=-1$, $d_{n}^{(r)}(-1)=d_{n}^{(r)}$ are the derangement numbers of order *r*.

For $0 \leq r \leq n$, the *r*-derangement numbers, denoted by $D_{n}^{(r)}$, are the number of derangements on $n+r$ elements under the restriction that the first *r*-elements are in disjoint cycles. It is known that the generating function of the *r*-derangement numbers is given by

2.23 $$ \begin{aligned}[b] \sum_{n=0}^{\infty}D_{n}^{(r)} \frac{t^{n}}{n!} = \frac{t^{r}}{(1-t)^{r+1}}e^{-t}. \end{aligned} $$

We consider the *r*-derangement polynomials given by

2.24 $$ \begin{aligned}[b] \frac{t^{r}}{(1-t)^{r+1}}e^{xt} = \sum_{n=0}^{\infty}D_{n}^{(r)}(x) \frac {t^{n}}{n!}\quad(0 \leq r \leq n). \end{aligned} $$

From (2.24), we note that $D_{n}^{(r)}(-1)=D_{n}^{(r)}$ are the *r*-derangement numbers. By (2.13) and (2.22), we easily get

2.25 $$ \begin{aligned}[b] d_{n}^{(r)}(x) \sim \bigl( (1-t)^{r},t\bigr) \quad(n \geq0) \end{aligned} $$

and

2.26 $$ \begin{aligned}[b] t^{r} d_{n}^{(r)}(x) = (n)_{r} d_{n-r}^{(r)}(x) = r! {n \choose r} d_{n-r}^{(r)}(x). \end{aligned} $$

From (2.22) and (2.24), we have

2.27 $$ \begin{aligned}[b] \sum_{n=0}^{\infty}D_{n}^{(r)}(x) \frac{t^{n}}{n!} &= t^{r} \sum _{n=0}^{\infty}d_{n}^{(r+1)}(x) \frac{t^{n}}{n!} \\ &= \sum_{n=r}^{\infty}{n \choose r} r! d_{n-r}^{(r+1)}(x) \frac{t^{n}}{n!}. \end{aligned} $$

Comparing the coefficients on both sides of (2.27), we get

2.28 $$ \begin{aligned}[b] D_{n}^{(r)}(x) = {n \choose r} r! d_{n-r}^{(r+1)}(x) \quad(n \geq r). \end{aligned} $$

From (2.22), we have

2.29 $$ \begin{aligned}[b] \sum_{n=0}^{\infty}d_{n}^{(r)}(0) \frac{t^{n}}{n!} = \biggl( \frac {1}{1-t} \biggr)^{r} = \sum_{n=0}^{\infty}{n+r-1 \choose n} t^{n} = \sum_{n=0}^{\infty}(n+r-1)_{n} \frac{t^{n}}{n!}. \end{aligned} $$

Thus, by (2.29), we get

2.30 $$ \begin{aligned}[b] d_{n}^{(r)}(0) = (n+r-1)_{n} \quad(n \geq0). \end{aligned} $$

From (2.22) and (2.24), we have

2.31 $$ \begin{aligned}[b] t^{r} \sum _{n=0}^{\infty}d_{n}^{(r+1)}(x) \frac{t^{n}}{n!} & = \frac {t^{r}}{(1-t)^{r+1}}e^{-t} e^{(x+1)t} \\ &= \Biggl( \sum_{l=0}^{\infty}D_{l}^{(r)} \frac{t^{l}}{l!} \Biggr) \Biggl( \sum _{m=0}^{\infty}(x+1)^{m}\frac{t^{m}}{m!} \Biggr) \\ &= \sum_{n=0}^{\infty}\Biggl( \sum _{l=0}^{n} {n \choose l} D_{l}^{(r)} (x+1) ^{n-l} \Biggr) \frac{t^{n}}{n!}. \end{aligned} $$

Therefore, by (2.27) and (2.31), we obtain the following theorem.

### Theorem 2.6

*For* $n \geq r$, *we have*

$$ \begin{aligned}[b] \sum_{l=0}^{n} {n \choose l} D_{l}^{(r)} (x+1) ^{n-l} = {n \choose r} r! d_{n-r}^{(r+1)}(x). \end{aligned} $$

Now, we observe that

2.32 $$ \begin{aligned}[b] \frac{t^{r-1}}{(1-t)^{r}} e^{xt} + \frac{t^{r}}{(1-t)^{r+1}} e^{xt} &= \frac{t^{r-1}}{(1-t)^{r+1}}e^{xt} \\ &= \biggl( \frac{t^{r-1}}{(1-t)^{r}} e^{-t} \biggr) \biggl( \frac {1}{1-t} e^{-t} \biggr) e^{(x+2)t} \\ &= \sum_{n=0}^{\infty}\Biggl( \sum _{k=0}^{n} \sum_{l=0}^{k} {n \choose k} {k \choose l} D_{l}^{(r-1)} d_{k-l}(x+2)^{n-k} \Biggr) \frac{t^{n}}{n!}. \end{aligned} $$

On the other hand, by (2.24), we get

2.33 $$ \begin{aligned}[b] \frac{t^{r-1}}{(1-t)^{r}} e^{xt} + \frac{t^{r}}{(1-t)^{r+1}} e^{xt} &= \sum_{n=0}^{\infty}\bigl( D_{n}^{(r-1)}(x) + D_{n}^{(r)}(x) \bigr) \frac{t^{n}}{n!}. \end{aligned} $$

From (2.32) and (2.33), we have

2.34 $$ \begin{aligned}[b] D_{n}^{(r-1)}(x) + D_{n}^{(r)}(x) = \sum_{k=0}^{n} \sum_{l=0}^{k} {n \choose k} {k \choose l} D_{l}^{(r-1)} d_{k-l}(x+2)^{n-k}. \end{aligned} $$

In particular, for $x=-1$, we get

2.35 $$ \begin{aligned}[b] D_{n}^{(r-1)} + D_{n}^{(r)} = \sum_{k=0}^{n} \sum_{l=0}^{k} {n \choose k} {k \choose l} D_{l}^{(r-1)} d_{k-l}. \end{aligned} $$

Therefore, by (2.34) and (2.35), we obtain the following theorem.

### Theorem 2.7

*For* $n \geq0$, *we have*

$$ \begin{aligned}[b] D_{n}^{(r-1)}(x) + D_{n}^{(r)}(x) = \sum_{k=0}^{n} \sum_{l=0}^{k} {n \choose k} {k \choose l} D_{l}^{(r-1)} d_{k-l}(x+2)^{n-k}. \end{aligned} $$

*Moreover*,

$$ \begin{aligned}[b] D_{n}^{(r-1)} + D_{n}^{(r)} = \sum_{k=0}^{n} \sum _{l=0}^{k} {n \choose k} {k \choose l} D_{l}^{(r-1)} d_{k-l}. \end{aligned} $$

By (2.22), we easily get

2.36 $$ \begin{aligned}[b] \sum_{n=0}^{\infty}d_{n}^{(r)}(x) \frac{t^{n}}{n!} &= \biggl( \frac {1}{1-t} \biggr)^{r} e^{-t} e^{(x+1)t} = \Biggl( \sum _{l=0}^{\infty}d_{l}^{(r)} \frac{t^{l}}{l!} \Biggr) \Biggl( \sum_{m=0}^{\infty}(x+1)^{m}\frac{ t^{m}}{m!} \Biggr) \\ &= \sum_{n=0}^{\infty}\Biggl(\sum _{l=0}^{n} {n \choose l} d_{l}^{(r)} (x+1)^{n-l} \Biggr) \frac{t^{n}}{n!}. \end{aligned} $$

Comparing the coefficients on both sides of (2.36), we have

2.37 $$ \begin{aligned}[b] d_{n}^{(r)}(x)= \sum _{l=0}^{n} {n \choose l} d_{l}^{(r)} (x+1)^{n-l}, \end{aligned} $$

with the usual convention about replacing $(d^{(r)})^{l}$ by $d_{l}^{(r)}$. Thus, by (2.37), we get

2.38 $$ \begin{aligned}[b] d_{n}^{(r)}(x+y) &= \bigl(d^{(r)}+x+y+1\bigr)^{n} = \bigl(d^{(r)}+x+1+y \bigr)^{n} \\ &= \sum_{l=0}^{n} {n \choose l} \bigl(d^{(r)}+x+1\bigr)^{l} y^{n-l} = \sum _{l=0}^{n} {n \choose l} d_{l}^{(r)}(x) y^{n-l}\quad(n \geq0). \end{aligned} $$

From (2.22), we can derive the following equation:

2.39 $$ \begin{aligned}[b] \sum_{n=0}^{\infty}d_{n}^{(r)}(x)\frac{t^{n}}{n!} &= \biggl( \frac {1}{1-t} \biggr)^{r} e^{xt} = \biggl( \frac{1}{1-t} \biggr)^{r-1} e^{-t} \biggl( \frac{1}{1-t} \biggr) e^{(x+1)t} \\ &= \Biggl(\sum_{l=0}^{\infty}d_{l}^{(r-1)} \frac{t^{l}}{l!} \Biggr) \Biggl(\sum _{m=0}^{\infty}d_{m}(x+1) \frac{t^{m}}{m!} \Biggr) \\ &=\sum_{n=0}^{\infty}\Biggl(\sum _{l=0}^{n} {n \choose l} d_{l}^{(r-1)} d_{n-l}(x+1) \Biggr)\frac{t^{n}}{n!}. \end{aligned} $$

Thus, by (2.39), we get

2.40 $$ \begin{aligned}[b] d_{n}^{(r)}(x) = \sum_{l=0}^{n} {n \choose l} d_{l}^{(r-1)} d_{n-l}(x+1)\quad(n \geq0). \end{aligned} $$

For $x=-2$, from (2.37) and (2.40) we have

2.41 $$ \begin{aligned}[b] d_{n}^{(r)}(-2) &= \sum_{l=0}^{n} {n \choose l} d_{l}^{(r-1)} d_{n-l} \\ &= \sum_{l=0}^{n} {n \choose l} d_{l}^{(r)}(-1)^{n-l}\quad(n \geq0). \end{aligned} $$

From (2.17), we have

2.42 $$ \begin{aligned}[b] \frac{e^{t}-1}{t} d_{n}^{(r)}(x) &= \int_{x}^{x+1} d_{n}^{(r)}(u) \,du = \frac {1}{n+1} \bigl\{ d_{n+1}^{(r)}(x+1) - d_{n+1}^{(r)}(x) \bigr\} \\ &= \frac{1}{n+1} \Biggl\{ \sum_{l=0}^{n+1} {n+1 \choose l} d_{l}^{(r)}(x) - d_{n+1}^{(r)}(x) \Biggr\} \\ &= \frac{1}{n+1} \sum_{l=0}^{n} {n+1 \choose l} d_{l}^{(r)}(x) \\ &= \frac{1}{n+1} \sum_{l=1}^{n+1} {n+1 \choose l} d_{n+1-l}^{(r)}(x)\quad(n \geq0). \end{aligned} $$

By (2.37) and (2.42), we get

2.43 $$ \begin{aligned}[b] d_{n}^{(r)}(x) &= \frac{1}{n+1} \sum_{l=1}^{n+1} \sum _{m=0}^{n+1-l} {n+1 \choose l} {n+1-l \choose m} d_{m}^{(r)} \frac{t}{e^{t}-1} (x+1)^{n+1-l-m} \\ &=\frac{1}{n+1} \sum_{l=1}^{n+1} \sum _{m=0}^{n+1-l} {n+1 \choose l} {n+1-l \choose m} d_{m}^{(r)}B_{m+1-l-m}(x+1). \end{aligned} $$

Therefore, by (2.43), we obtain the following theorem.

### Theorem 2.8

*For* $n \geq0$, *we have*

$$ \begin{aligned}[b] d_{n}^{(r)}(x) = \frac{1}{n+1} \sum_{l=1}^{n+1} \sum _{m=0}^{n+1-l} {n+1 \choose l} {n+1-l \choose m} d_{m}^{(r)}B_{m+1-l-m}(x+1). \end{aligned} $$

For $n \geq0$, let

$$ \begin{aligned}[b] \mathbb{P}_{n}= \bigl\{ p(x) \in \mathbb{C}[x] \vert \deg p(x) \leq n \bigr\} . \end{aligned} $$

Then $\mathbb{P}_{n}$ is an $(n+1)$-dimensional vector space over $\mathbb{C}$.

For $p(x) \in\mathbb{P}_{n}$, we let

2.44 $$ \begin{aligned}[b] p(x) = \sum _{k=0}^{n} C_{k} d_{k}(x). \end{aligned} $$

From (1.4), we have

2.45 $$ \begin{aligned}[b] \bigl\langle (1-t)t^{k} \vert p(x)\bigr\rangle &= \sum_{l=0}^{n} C_{l} \bigl\langle (1-t)t^{k} \vert d_{l}(x)\bigr\rangle \\ &= \sum_{l=0}^{n} C_{l} l! \delta_{k,l} = C_{k} k! \quad(k \geq0). \end{aligned} $$

Thus, we have

2.46 $$ \begin{aligned}[b] C_{k} = \frac{1}{k!} \bigl\langle (1-t)t^{k} \vert p(x)\bigr\rangle = \frac {1}{k!} \bigl\langle (1-t) \vert p^{(k)}(x)\bigr\rangle . \end{aligned} $$

Therefore, by (2.44) and (2.46), we obtain the following theorem.

### Theorem 2.9

*For* $p(x) \in\mathbb{P}_{n}$, *we have*

$$ \begin{aligned}[b] p(x) = \sum_{k=0}^{n} C_{k} d_{k}(x), \end{aligned} $$

*where* $C_{k} = \frac{1}{k!} \langle (1-t)t^{k}|p(x)\rangle = \frac {1}{k!} \langle(1-t)|p^{(k)}(x)\rangle$.

Let us take $p(x) = d_{n}^{(r)}(x) \in\mathbb{P}_{n}$. Then we have

2.47 $$ \begin{aligned}[b] p(x) = \sum _{l=0}^{n} C_{l} d_{l}(x), \end{aligned} $$

where

2.48 $$ \begin{aligned}[b] C_{l} &= \frac{1}{l!} \bigl\langle 1-t \vert p^{(l)}(x) \bigr\rangle = \frac {1}{l!} \bigl\langle 1-t \vert(n)_{l} d_{n-l}^{(r)}(x)\bigr\rangle \\ &= {n \choose l} \bigl\langle 1-t \vert d_{n-l}^{(r)}(x) \bigr\rangle = {n \choose l} d_{n-l}^{(r)}(0) - {n \choose l+1} (l+1) d_{n-l-1}^{(r)}(0). \end{aligned} $$

Hence, by (2.47) and (2.48), we get

$$ \begin{aligned}[b] d_{n}^{(r)}(x)= \sum _{l=0}^{n} \biggl\{ {n \choose l} d_{n-l}^{(r)}(0) - {n \choose l+1} (l+1) d_{n-l-1}^{(r)}(0) \biggr\} d_{l}(x). \end{aligned} $$

Assume that $p(x) = \sum_{k=0}^{n} C_{k}^{(r)}d_{k}^{(r)}(x) \in\mathbb {P}_{n}$. Then, by (2.25), we get

2.49 $$ \begin{aligned}[b] \bigl\langle (1-t)^{r} t^{k} \vert p(x) \bigr\rangle &= \sum_{l=0}^{n} C_{l}^{(r)} \bigl\langle (!-t)^{r} t^{k} \vert d_{l}^{(r)}(x)\bigr\rangle \\ &= \sum_{l=0}^{n} C_{l}^{(r)}l! \delta_{l,k} = C_{k}^{(r)}k!\quad(k \geq0). \end{aligned} $$

Thus, from (2.49), we note that

2.50 $$ \begin{aligned}[b] C_{k}^{(r)} = \frac{1}{k!} \bigl\langle (1-t)^{r} t^{k} \vert p(x) \bigr\rangle =\frac{1}{k!} \bigl\langle (1-t)^{r} \vert p^{(k)}(x)\bigr\rangle . \end{aligned} $$

Therefore, we obtain the following theorem.

### Theorem 2.10

*For* $n \geq0$, *we have*

$$ \begin{aligned}[b] p(x) = \sum_{k=0}^{n} C_{k}^{(r)} d_{k}^{(r)}(x) \in \mathbb{P}_{n}, \end{aligned} $$

*where*

$$ \begin{aligned}[b] C_{k} = \frac{1}{k!} \bigl\langle (1-t)^{r} t^{k} \vert p(x)\bigr\rangle = \frac {1}{k!} \bigl\langle (1-t)^{r} \vert p^{(k)}(x)\bigr\rangle . \end{aligned} $$

### Example 1

For $p(x)=d_{n}(x)\in\mathbb{P}_{n}$, we have

$$ \begin{aligned}[b] p(x) = \sum_{k=0}^{n} C_{k}^{(r)}d_{k}^{(r)}(x), \end{aligned} $$

where

$$ \begin{aligned}[b] C_{k}^{(r)} &= \frac{1}{k!} \bigl\langle (1-t)^{r} \vert p^{(k)}(x) \bigr\rangle = \binom{n}{k}\bigl\langle (1-t)^{r} \vert d_{n-k}(x) \bigr\rangle \\ &= {n \choose k} \sum_{j=0}^{r} {r \choose j} (-1)^{j} \bigl\langle t^{j} \vert d_{n-k}(x)\bigr\rangle = {n \choose k} \sum _{j=0}^{r} {r \choose j} (-1)^{j} (n-k)_{j} d_{n-k-j}(0) \\ &= {n \choose k} \sum_{j=0}^{r} {r \choose j} (-1)^{j} (n-k)_{j}(n-k-j)! = {n \choose k} \sum_{j=0}^{r} {r \choose j} (-1)^{j} (n-k)!. \end{aligned} $$

Thus, we note that

$$ \begin{aligned}[b] d_{n}(x) = \sum _{k=0}^{n} \Biggl( \sum_{j=0}^{r} {n \choose k} (n-k)! {r \choose j} (-1)^{j} \Biggr) d_{k}^{(r)}(x). \end{aligned} $$

### Example 2

For $p(x)=B_{n}(x)$ ($n \geq0$), we have

$$ \begin{aligned}[b] B_{n}(x) = \sum _{k=0}^{n} C_{k}^{(r)}d_{k}^{(r)}(x), \end{aligned} $$

where

$$ \begin{aligned}[b] C_{k}^{(r)} &= \frac{1}{k!} \bigl\langle (1-t)^{r} t^{k} \vert B_{n}(x) \bigr\rangle = {n \choose k} \bigl\langle (1-t)^{r} \vert B_{n-k}(x)\bigr\rangle \\ &={n \choose k} \sum_{j=0}^{r} {r \choose j} (-1)^{j} \bigl\langle t^{j} \vert B_{n-k}(x)\bigr\rangle \\ &= {n \choose k} \sum_{j=0}^{r} {r \choose j} (-1)^{j} (n-k)_{j} \bigl\langle 1 \vert B_{n-k-j}(x)\bigr\rangle \\ &= {n \choose k} \sum_{j=0}^{r} {r \choose j} (-1)^{j} {n-k \choose j} j! B_{n-k-j}. \end{aligned} $$

Hence

$$ \begin{aligned}[b] B_{n}(x) = \sum _{k=0}^{n} \Biggl(\binom{n}{k}\sum _{j=0}^{r} {r \choose j} (-1)^{j} {n-k \choose j} j! B_{n-k-j} \Biggr) d_{k}^{(r)}(x). \end{aligned} $$

### Example 3

For $p(x)=E_{n}(x)\in\mathbb{P}_{n}$ ($n \geq0$), we have

$$ \begin{aligned}[b] E_{n}(x) = \sum _{k=0}^{n} C_{k}^{(r)}d_{k}^{(r)}(x), \end{aligned} $$

where

$$ \begin{aligned}[b] C_{k}^{(r)} &= \frac{1}{k!} \bigl\langle (1-t) t^{k} \vert E_{n}(x) \bigr\rangle = {n \choose k} \bigl\langle (1-t) \vert E_{n-k}(x)\bigr\rangle \\ &={n \choose k} E_{n-k}-{n \choose k} (n-k)E_{n-k-1}. \end{aligned} $$

Thus, we get

$$ \begin{aligned}[b] E_{n}(x) = \sum _{k=0}^{n} \biggl\{ {n \choose k} E_{n-k}-{n \choose k} (n-k)E_{n-k-1} \biggr\} d_{k}^{(r)}(x). \end{aligned} $$

### Example 4

For $p(x)=\mathrm{Bel}_{n}(x)\in\mathbb{P}_{n}$, we have

$$ \begin{aligned}[b] \mathrm{Bel}_{n}(x) = \sum _{k=0}^{n} C_{k}^{(r)}d_{k}^{(r)}(x), \end{aligned} $$

where

$$ \begin{aligned}[b] C_{k}^{(r)} &= \frac{1}{k!} \bigl\langle (1-t) t^{k}\vert \mathrm{Bel}_{n}(x) \bigr\rangle = \frac{1}{k!} \Biggl\langle (1-t)t^{k} \Big\vert \sum _{m=0}^{n} S_{2}(n,m)x^{m}\Biggr\rangle \\ &= \sum_{m=k}^{n} S_{2}(n,m) {m \choose k} \bigl\langle (1-t) \vert x^{m-k}\bigr\rangle \\ &= S_{2}(n,k) - \sum_{m=k}^{n} S_{2}(n,m) {m \choose k} (m-k) 0^{m-k-1} \\ &= S_{2}(n,k) - S_{2}(n,k+1) (k+1) = 2S_{2}(n,k) - S_{2}(n+1,k+1). \end{aligned} $$

Hence

$$ \begin{aligned}[b] \mathrm{Bel}_{n}(x) = \sum _{k=0}^{n} \bigl( 2S_{2}(n,k) - S_{2}(n+1,k+1) \bigr) d_{k}^{(r)}(x). \end{aligned} $$

The ordered Bell polynomials are defined by the generating function

2.51 $$ \begin{aligned}[b] \frac{1}{2-e^{t}} e^{xt} = \sum_{n=0}^{\infty}b_{n}(x) \frac{t^{n}}{n!}. \end{aligned} $$

When $x=0$, $b_{n}=b_{n}(0)$ ($n \geq0$) are the ordered Bell numbers. From (2.12) and (2.51), we note that $b_{n}(x) \sim (2-e^{t},t)$ ($n \geq0$). For $b_{n}(x) \sim(2-e^{t},t)$, $d_{n}(x) \sim(1-t,t)$, by (2.7) and (2.13), we get

2.52 $$ \begin{aligned}[b] b_{n}(x) = \sum _{m=0}^{n} C_{n,m}d_{m}(x)\quad(n \geq0), \end{aligned} $$

where

2.53 $$ \begin{aligned}[b] C_{n,m} &= \frac{1}{m!} \biggl\langle \frac{1-t}{2-e^{t}} t^{m} \Big\vert x^{n} \biggr\rangle = {n \choose m} \biggl\langle \frac{1-t}{2-e^{t}}\Big\vert x^{n-m} \biggr\rangle \\ &= {n \choose m} \biggl\langle 1-t \Big\vert \frac{1}{2-e^{t}}x^{n-m} \biggr\rangle = {n \choose m} \bigl\langle 1-t \vert b_{n-m}(x)\bigr\rangle \\ &= {n \choose m} \bigl\{ b_{n-m} - (n-m) b_{n-m-1} \bigr\} . \end{aligned} $$

Therefore, we obtain the following theorem.

### Theorem 2.11

*For* $n \geq0$, *we have*

$$ \begin{aligned}[b] b_{n}(x) = \sum _{m=0}^{n} {n \choose m} \bigl( b_{n-m} - (n-m) b_{n-m-1} \bigr) d_{m}(x). \end{aligned} $$

For $d_{n}(x) \sim(1-t,t)$, $(x)_{n} \sim(1,e^{t}-1)$, we have

2.54 $$ \begin{aligned}[b] d_{n}(x) = \sum _{m=0}^{n} C_{n,m} (x)_{m}\quad(n \geq0), \end{aligned} $$

where

2.55 $$ \begin{aligned}[b] C_{n,m} &= \frac{1}{m!} \biggl\langle \frac{1}{1-t}\bigl(e^{t}-1\bigr)^{m} \Big\vert x^{n} \biggr\rangle = \sum_{l=m}^{n} S_{2}(l,m) \frac{1}{l!} \biggl\langle \frac{t^{l}}{1-t} \Big\vert x^{n} \biggr\rangle \\ &= \sum_{l=m}^{n} S_{2}(l,m) {n \choose l} \biggl\langle \frac{1}{1-t} \Big\vert x^{n-l} \biggr\rangle = \sum_{l=m}^{n} S_{2}(l,m) {n \choose l} \bigl\langle 1 \vert d_{n-l}(x)\bigr\rangle \\ &= \sum_{l=m}^{n} S_{2}(l,m) {n \choose l} d_{n-l}(0). \end{aligned} $$

Therefore, by (2.54) and (2.55), we obtain the following theorem.

### Theorem 2.12

*For* $n \geq0$, *we have*

$$ \begin{aligned}[b] d_{n}(x) = \sum _{m=0}^{n} \Biggl( \sum_{l=m}^{n} S_{2}(l,m) {n \choose l}d_{n-l}(0) \Biggr) (x)_{m}. \end{aligned} $$

## Results and discussion

In this paper, as a natural companion to derangement numbers, we have investigated derangement polynomials and derived several interesting properties on them which are related to derangement numbers. Also, we have considered two generalizations of derangement polynomials, namely the higher-order and *r*-derangement polynomials, and showed some relations between them and also with some other special polynomials. In addition, by using umbral calculus, we derived a formula expressing any polynomials as linear combinations of higher-order derangement polynomials and illustrated this with several special polynomials.

## Conclusion

The introduction of derangement numbers goes back to as early as 1708 when Pierre Rémond de Montmort considered some counting problem on derangements. However, it seems that the umbral calculus approach to the derangement polynomials and their generalizations has not yet been done. In this paper, we have used umbral calculus in order to study some interesting properties on them, certain relations between them, and some connections with several other special polynomials.
